# Gas Station Heroin: A Case Report of Tianeptine Use Disorder and a Literature Review

**DOI:** 10.7759/cureus.95153

**Published:** 2025-10-22

**Authors:** Kalyan Kandra, Raghavendran Gajagowni, Gabriela A Fernandez, Natalie Polechonski, Brandon P Blewett

**Affiliations:** 1 Psychiatry, Southern Illinois University School of Medicine, Springfield, USA; 2 Psychiatry, North Mississippi Medical Center, Tupelo, USA

**Keywords:** addiction, antidepressant, gas station heroin, nootropic, opioid agonist, opioid use disorders, substance use disorder, tianeptine, tianna red, zaza

## Abstract

Tianeptine, an atypical antidepressant prescribed in several countries, is not approved for medical use in the United States. Despite this, it is widely available as an unregulated supplement, popularly known as “gas station heroin,” contributing to a growing public health crisis characterized by dependence, severe withdrawal, and overdose. We present the case of a 22-year-old male with major depressive disorder who developed severe tianeptine use disorder. He presented with worsening depression and suicidal ideation, with his psychiatric symptoms substantially exacerbated by cycles of intoxication and withdrawal. The patient declined referral to medication-assisted treatment, highlighting ongoing barriers to care. This case underscores diagnostic challenges when psychiatric symptoms mask underlying substance use disorders and emphasizes the urgent need for clinician awareness, patient education, and regulatory action.

## Introduction

Tianeptine is considered an atypical antidepressant due to its distinct pharmacological profile that differs from traditional monoaminergic agents [1]. It has since been identified as a potent µ-opioid receptor agonist, a property responsible for its abuse potential [2]. In the United States, its misuse has been documented under the street names "Gas station heroin," “ZaZa,” and “Tianna Red” [3]. Reports describe escalating patterns of abuse and dependence, particularly at supratherapeutic doses [4]. Neuropsychiatric complications such as psychosis have also been observed in individuals misusing tianeptine [5]. Reviews of clinical cases emphasize the high risk of dependence in psychiatric patients [6].

Earlier international systematic reviews established tianeptine’s antidepressant efficacy [7]. Nursing literature underscores the importance of clinician awareness of its misuse [8]. Experimental studies revealed its role in neuroplasticity, linking its effects to glutamatergic modulation [9]. Regulatory discussions highlight gaps in oversight that have permitted wide availability [10]. Its therapeutic benefits in depression, including coexisting anxiety disorders, were established in early pharmacological reviews [11]. More recently, case reports have demonstrated characteristic withdrawal symptoms that mimic opioid withdrawal [12]. Clinical trials confirmed its benefit in melancholic depression [13]. Neurobiological reviews support its glutamatergic mechanism and effects on stress pathways [14].

Epidemiologic reports illustrate the scope of the public health crisis. A Tennessee surveillance study reported sharp increases in emergency department visits, overdoses, and seizures between 2021 and 2023 [15]. Other clinical updates echo these findings. Nursing commentaries stress the vulnerability of patients with opioid use disorder to misuse tianeptine. National poison center data confirmed rising exposures from 2015 to 2023 [16].

## Case presentation

A 22-year-old male with a history of major depressive disorder presented to the emergency department reporting a one-month history of worsening depressive symptoms. These included low mood, loss of interest, excessive sleeping, hopelessness, and worthlessness, which had culminated in active suicidal ideation for one day prior to his arrival. The patient was first diagnosed with depression two years ago, an onset that predated his tianeptine use. His past medication trials include bupropion XL 300 mg, which he took for two months before self-discontinuing, and citalopram 40 mg, which he had been taking for the past three months without notable improvement.

The patient disclosed approximately one year of nonmedical tianeptine use. He initially began using it to self-medicate for chronic pain after his prescribed hydrocodone was discontinued. His use escalated rapidly from small amounts to several grams per day, purchased from local gas stations, to sustain a short-lived euphoria and avoid withdrawal. Multiple attempts to quit had failed due to severe withdrawal symptoms he described as "worse than opioid withdrawal," which included anxiety, muscle pain, sweating, and gastrointestinal distress.

He was admitted to the inpatient psychiatric unit for stabilization. During admission, he developed mild withdrawal symptoms of diarrhea and anxiety that were managed conservatively with loperamide and as-needed hydroxyzine 25 mg. Fluoxetine 20 mg daily was initiated, leading to the improvement of his mood and resolution of suicidal ideation within two days. The diagnosis of tianeptine use disorder (severe) was made based on his compulsive use, dose escalation, inability to quit despite harm, and a withdrawal syndrome consistent with DSM-5-TR criteria [17]. Despite counseling, he declined a referral to a medication-assisted treatment (MAT) clinic. The patient was discharged on day 5 and was prescribed fluoxetine 20 mg once daily with a scheduled follow-up appointment at the outpatient hospital clinic in two weeks.

Laboratory findings on admission were significant for a mild leukocytosis with neutrophilia, elevated liver enzymes, and severe vitamin D deficiency. The urine toxicology screen was positive for cannabinoids. The clinical course is outlined in Table 1, and detailed laboratory results are presented in Table 2.

**Table 1:** Clinical course and timeline of tianeptine use ED, emergency department; MAT, medication-assisted treatment

Time Point	Event/Medication Change	Clinical Observation/Patient Report
~1 year prior	Opioid prescription discontinued	Began self-medicating with tianeptine
Past year	Escalating tianeptine use	Increased to multiple grams/day to maintain effects
Weeks prior to admission	Multiple cessation attempts	Unable to quit due to severe withdrawal
Presentation	ED visit	Worsening depression and suicidal ideation
Admission day 1	Inpatient admission	Mild withdrawal; managed conservatively
During admission	Started fluoxetine 20 mg daily	Mood improved; suicidal ideation resolved
Discharge	Declined MAT referral	Elevated relapse risk post-discharge

**Table 2:** Laboratory findings on admission

Laboratory Test	Result	Flag
Complete blood count		
Hemoglobin	14.5 g/dL	-
Hematocrit	42%	-
RBC	4.59 M/uL	Low
WBC	10.7 K/uL	High
Neutrophils	73%	High
Lymphocytes	19%	Low
Platelets	370 K/uL	-
Routine chemistries and enzymes		
Sodium	136 mmol/L	-
Potassium	3.7 mmol/L	-
Chloride	108 mmol/L	High
Anion gap	4 mmol/L	Low
Glucose	109 mg/dL	High
AST (SGOT)	49 U/L	High
ALT (SGPT)	92 U/L	High
Miscellaneous labs		
Vitamin D, 25-hydroxy	<7.0 ng/mL	Low
Toxicology		
Cannabinoids screen	Positive (unconfirmed)	-

## Discussion

This case illustrates several important themes in the clinical and public health understanding of tianeptine use disorder. Case series and systematic reviews highlight the overlap between psychiatric comorbidity and misuse, suggesting that vulnerable populations may be at particularly high risk [6]. The patient’s presentation of worsening depression and suicidality, in the context of escalating tianeptine use, exemplifies how substance use can exacerbate underlying psychiatric illness and obscure accurate diagnosis.

Pharmacologically, tianeptine occupies a unique position. Initially developed as an antidepressant with atypical mechanisms involving glutamatergic modulation and neuroplasticity [1,9,14], it was marketed as an alternative for patients who did not respond to traditional serotonergic agents. However, its potent µ-opioid receptor agonism, discovered later, is now recognized as the primary driver of its misuse [2]. At therapeutic doses, glutamatergic modulation may confer antidepressant benefits, but at supratherapeutic doses, opioid receptor activation predominates, leading to intoxication, tolerance, dependence, and withdrawal [2,4]. These pharmacological insights explain the severe clinical course of patients with misuse, including the opioid-like withdrawal syndrome, which has been successfully reversed in some cases with naloxone [12].

Neuropsychiatric sequelae of misuse are increasingly documented. In addition to dependence, case reports describe episodes of psychosis, agitation, and suicidality [4,5]. Our case highlights the diagnostic complexity, where depressive symptoms, rather than overt intoxication or withdrawal, brought the patient to clinical attention. This underscores the need for careful substance use screening in patients with refractory psychiatric presentations.

From a regulatory and systems perspective, the tianeptine crisis mirrors the broader opioid epidemic. Nursing and regulatory literature call attention to the “gas station heroin” phenomenon, where psychoactive substances are marketed under the guise of dietary supplements or nootropics, avoiding regulatory oversight [8,10,16]. Inconsistent regulation across states has contributed to ongoing availability. Meanwhile, clinicians may be unaware of its presence in their communities, compounding delays in recognition and treatment [3].

Epidemiological data strongly support the growing scope of harm. Poison center calls have risen steadily in recent years [16], and state-level surveillance has documented increases in overdoses and fatalities linked to tianeptine [15,16]. These findings align with case-based evidence and underscore the importance of clinician vigilance. To provide a broader context for this case, a targeted literature review on "tianeptine pharmacology and abuse" was conducted. This review encompassed various studies, including review of the pharmacology properties, mechanism of action, and abuse potential. Case report studies highlighted dependence and misuse patterns as well as the mortality and morbidity associated with tianeptine dependence and misuse (Table 3).

**Table 3:** Targeted literature review on tianeptine

Author (Year)	Study Type/Population	Key Findings
Alamo et al. (2019) [1]	Review	Atypical pharmacology; glutamatergic effects
Nishio et al. (2024) [2]	Neuroscience review	MOR agonism; abuse potential
Wagner et al. (2023) [3]	Narrative review	“Gas station heroin,” U.S. misuse, regulation
Lauhan et al. (2018) [4]	Case report	Withdrawal and misuse patterns
Karim and Ioannou (2020) [5]	Case report	Psychosis is associated with misuse
Springer and Cubała (2018) [6]	Case series review	Psychiatric comorbidities in dependence
Wagstaff et al. (2001) [7]	Review	Antidepressant efficacy
Wilde and Benfield (1995) [11]	Pharmacologic review	PK/PD, efficacy in depression/anxiety
Ginestet (1997) [13]	Clinical trial	Efficacy in melancholic depression
McEwen et al. (2010) [14]	Neurobiological review	Glutamatergic modulation
Uzbay (2008) [9]	Experimental review	Neuroplasticity changes
Hargett et al. (2023) [8]	Nursing review	Clinical implications of misuse
Hargett et al. (2024) 10]	Regulatory update	State restrictions and advisories
Farsani and Reyes (2024) [12]	Case report	Withdrawal and naloxone reversal
Hershey et al. (2024) [15]	Surveillance	Tennessee ED visits and deaths
Quadir et al. (2025) [16]	Toxicology study	National poison center data

While no FDA-approved medications exist specifically for tianeptine use disorder, clinical strategies are adapted from evidence-based treatments for opioid use disorder, reflecting tianeptine's primary action as a µ-opioid receptor agonist. The management of acute withdrawal often involves symptomatic care, though buprenorphine has proven effective for more severe cases. For long-term treatment, MAT with opioid agonists such as buprenorphine or methadone is considered the standard of care to reduce cravings and prevent relapse, highlighting the significance of this patient's refusal of a MAT referral. Given the risk of respiratory depression, naloxone is a critical intervention for overdose reversal. These pharmacological approaches are most effective when integrated with comprehensive psychosocial support, such as counseling and behavioral therapies, to address the full scope of the disorder.

The diagnostic complexity highlighted by this case underscores the need for greater clinical vigilance. Clinicians must maintain a high index of suspicion for tianeptine use disorder, particularly when patients present with atypical or treatment-refractory depressive and anxiety symptoms. Also, the recurrent cycle of tianeptine intoxication and withdrawal can precipitate or significantly exacerbate depressive episodes, making it difficult to distinguish from an underlying mood disorder. This clinical challenge is situated within a growing public health crisis, as evidenced by rising poison center calls and state-level reports of overdose and death, which signal an urgent need for a coordinated response. Addressing the harms of tianeptine will require a multi-faceted approach including improved provider awareness and enhanced regulatory oversight.

Addressing the tianeptine crisis requires urgent policy action and targeted research. Enhanced federal and state regulatory oversight is needed to curb its availability as an unregulated supplement, supported by robust public health surveillance and clinician education campaigns. Future research should prioritize clinical trials to establish evidence-based treatment protocols, particularly for MATs such as buprenorphine. Additionally, longitudinal studies are needed to clarify long-term outcomes, and new screening tools must be developed to help clinicians identify misuse earlier in patients presenting with complex psychiatric symptoms.

## Conclusions

Tianeptine's potent µ-opioid receptor activity is the primary driver of its widespread misuse. The case presented demonstrates that tianeptine use disorder can clinically masquerade as worsening psychiatric illness, which significantly delays recognition of the underlying substance use disorder. This diagnostic challenge is exacerbated by systemic factors, including inconsistent regulation and the substance's commercial availability in retail settings, which have fueled a growing public health crisis. Addressing the tianeptine crisis requires improving clinician awareness and screening, enhancing regulatory oversight of supplements, strengthening public health surveillance, and expanding access to evidence-based treatment for tianeptine use disorder.
